# Measurement of wrist flexion and extension torques in different forearm positions

**DOI:** 10.1186/s12938-015-0110-9

**Published:** 2015-12-12

**Authors:** Yuichi Yoshii, Hiroshi Yuine, Ohashi Kazuki, Wen-lin Tung, Tomoo Ishii

**Affiliations:** Department of Orthopaedic Surgery, Tokyo Medical University Ibaraki Medical Center, 3-20-1 Chuo, Ami, Inashiki, Ibaraki 300-0395 Japan; Department of Rehabilitation, Ibaraki Prefectural University of Health Sciences, Ami, Ibaraki 300-0394 Japan

**Keywords:** Wrist torque, Forearm, Isometric, Epicondylitis

## Abstract

**Background:**

Forceful activities of the wrist were considered to be a risk factor for the epicondylitis. However, there are still conflicting evidence concerning work-relatedness of epicondylitis. The main problem is that there is little information about which forearm postures are capable of withstanding higher torque loads and the extent of the differences in the torques generated by different forearm postures. The objective of this study was to investigate the differences in wrist flexion and extension torques among different forearm positions in healthy subjects.

**Methods:**

Twenty wrists of 10 asymptomatic volunteers were evaluated. The apparatus to measure the wrist torque consisted of a handle with a force sensor and a table to place the forearm in different positions. The direction of the handle can change when measuring different forearm positions. The forearm of the examinee was secured to the table. The participants were asked to exert themselves in maximal isometric contraction for wrist flexion or extension, and to maintain it for 5 s. Each evaluation of the flexion and extension torque was conducted twice. Three forearm positions were evaluated: neutral, pronation, and supination. The intra-class correlation coefficients between first and second measurements were evaluated for the maximum torque. The maximum torques and flexion/extension ratio were compared among the positions. In addition, the agility and endurance for the wrist extension/flexion torques were compared among the positions.

**Results:**

The intra-class correlation coefficients between first and second measurements were 0.928 and 0.866 for the wrist flexion and extension measurements, respectively. The highest torques for the wrist flexion and extension were observed in the supination and pronation positions, respectively (P < 0.01). There was a higher extension/flexion ratio in the supination position compared to the other positions (P < 0.05). There was a superior agility for the wrist flexion in the supination position compared to the pronation position.

**Conclusions:**

The normal balance of the wrist flexion–extension torques in different forearm positions were characterized. This information might aid the provision of advice regarding the optimal positions for performing specific tasks and could help to elucidate the pathophysiology of epicondylitis.

## Background

Epicondylitis is one of the most prevalent disorders, with an estimated prevalence of 5 % in the general population [1]. Epicondylitis can be divided into lateral epicondylitis, known as tennis elbow, and medical epicondylitis, which is known as golfers elbow. Lateral/medial epicondylitis is considered to be a mechanical stress injury and occurs after minor (and often unrecognized) trauma to the proximal insertion of the extensor carpi radialis brevis muscle or the flexor–pronator musculature at the epicondyle of the humerus.

In previous studies, it was suggested that the epicondylitis caused by overload or repetitive stress at the tendon insertion with the specific workload [2–5]. There were statistically significant association between combinations of exposures (force, repetitiveness, and posture) and the occurrence of epicondylitis [5]. The arms lifted in front of the body, hands bent or twist, and precision movement during a part of the working day were considered to be risk factors for the lateral epicondylitis. In addition, handling loads and working with high hand grip forces were positively associated medial epicondylitis [1]. These studies presented that overview of the risk factors for the epicondylitis. However, there are still conflicting evidence concerning work-relatedness of epicondylitis. The main problem is that there is little information about which forearm postures are able to withstand higher torque loads and the extent of the differences between the torques generated by different forearm postures.

Pronation–supination of the forearm involves the rotation of the forearm around its longitudinal axis. This motion is important because it allows the hand to be oriented upwards or downwards. The stress placed on the forearm muscle insertion during such movements might be influenced by the interactions between the associated joints, muscles, and tendons. Since the application of muscle tension to a moment arm produces joint torque, alterations in forearm position might affect wrist torque. Torque ratios [6] and moment arm ratios [7] have previously been used as measures of wrist balance. In addition, the relationship between wrist position and strength/torque [8] has been examined. However, the relationships between forearm rotation and wrist torques are still unclear. If it were possible to clarify the wrist torques generated in different forearm positions, it would be helpful to understand how much workload specific postures can withstand. In addition, such information would aid the provision of advice regarding the optimal forearm positions for performing specific tasks. Thus, it is important to characterize the normal torque values for wrist flexion and extension in different forearm positions. The objective of this study was to investigate the differences in the wrist flexion and extension torques generated by different forearm positions in healthy subjects.

## Methods

The protocol for this study was reviewed and approved by our Institutional Review Board. The procedures followed were in accordance with the ethical standards of the responsible committee on human experimentation (institutional and national) and with the Helsinki Declaration for experiments involving humans. Twenty wrists of 10 asymptomatic volunteers (all men; age range 22–31, mean 25.5 years) were evaluated. All participants were right-handed, in good health, and free of musculoskeletal disorders of the upper extremities.

### Apparatus

To measure the wrist torques in different forearm positions, we developed an apparatus. The apparatus consisted of a handle to measure the wrist torque and a table to place the forearm in different positions (Three-One Design, Inc., Tsukuba, Japan). The handle was equipped with a torque sensor (UTMII-20Nm, Unipulse Co., Tokyo, Japan). The torque sensor has 1/10,000 Nm resolution, zero-point stability and sampling frequency of 6 Hz. The placement of the handle can be adjusted according to the anthropometric dimensions of the rotation center of the wrist for each participant. The direction of the handle can change when measuring different positions of the forearm, that is, neutral, pronation, and supination positions. The equipment can measure the torque in the range of 0–20 Nm. The torque delivered by the sensor was displayed on a monitor and recorded as text data. The apparatus allows measurement of the isometric and isokinetic torque.

### Wrist torque measurement

**Fig. 1:**
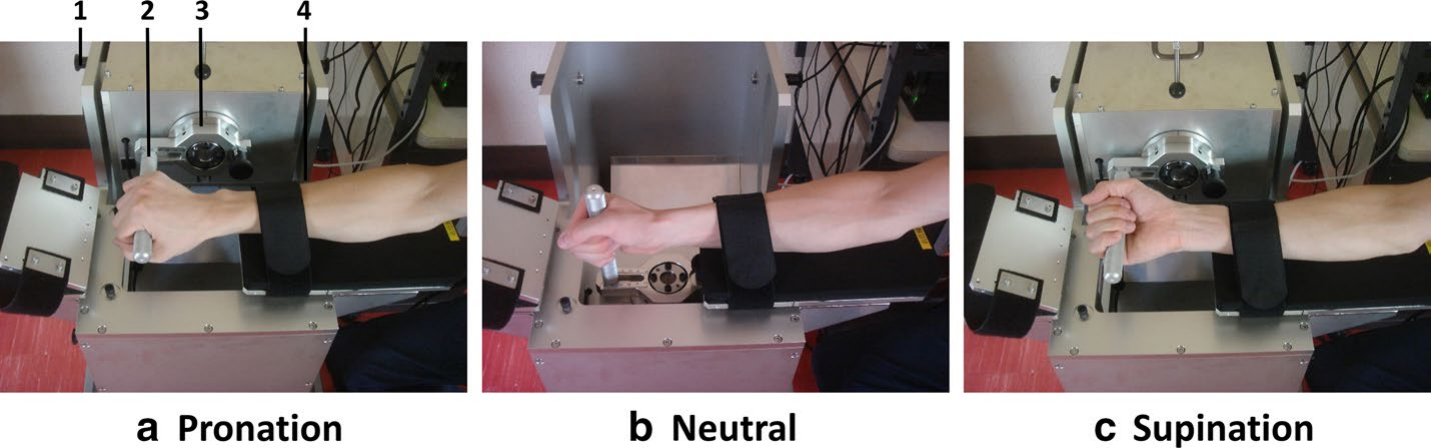
Experimental setup. The *pictures* showed each of the forearm positions. The apparatus to measure the wrist torque consisted of a handle to measure the wrist torque and a table to place the forearm. *1* Lock to change the direction of the handle, *2* handle, *3* torque sensor, *4* forearm table. **a** Pronation position. **b** Neutral position. **c** Supination position

The participants sat on a chair that could be adapted to individual anthropometric characteristics. Each subject was measured with an upper limb hung downward and the elbow flexed in a 45-degree position. The forearm of the examinee was secured to the forearm table with a bandage. To prevent the subjects using elbow joint torque, they were instructed to keep their forearm in contact with the table. Three forearm positions were retained for this study: neutral, pronation, and supination. Figure 1 shows the experimental setup for each position. To standardize the positions, the volar surface of the hand was maintained parallel to the handle.

The participants were asked to exert themselves in maximal voluntary contraction as quickly as possible, and to maintain it for 5 s. The handle position was adjusted so that the rotation center of the wrist fitted to the rotation center of the equipment, and fixed. The isometric torques in the wrist neutral position were evaluated. The flexion and extension torques were measured on different days. Each evaluation of the flexion and extension torque was conducted twice. The first flexion and extension torque measurements were obtained in the same week. One week after the first measurements, the second flexion and extension torque measurements were performed. Firstly, the positions were chosen randomly, followed by the other positions, for example, pronation–supination–neutral. Secondly, the measurement was started in a different position from the first time, followed by the other positions. Rest periods lasting a few minutes were allowed between trials.

### Data analysis

**Fig. 2:**
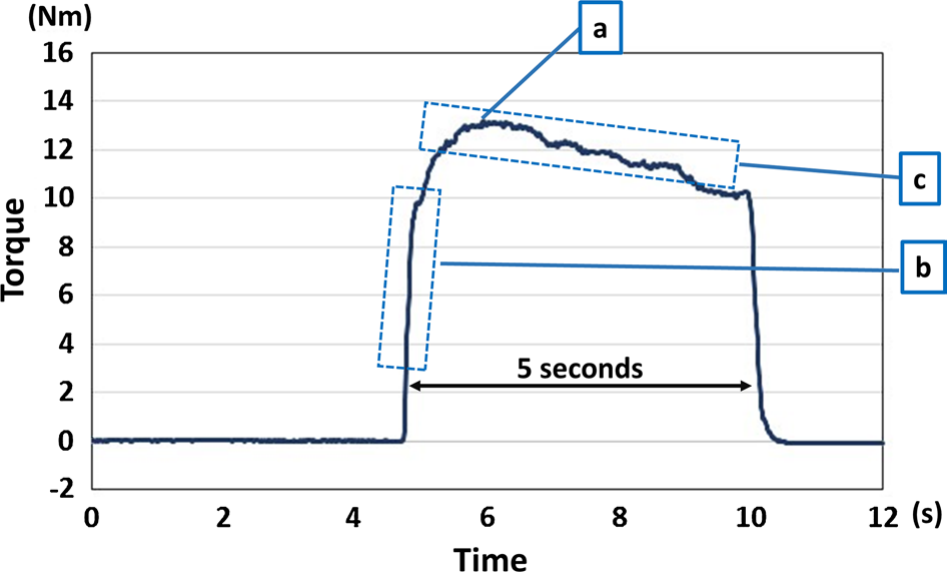
A sample of wrist torque waveform. **a** The maximum torque was chosen for the analysis. **b** The agility of the muscle strength was defined as the initial slope of the torque/time curve from 25 to 75 % of the maximum torque (Nm/ms). **c** The endurance was evaluated by the time (s) and the slope (Nm/ms) of the waveform period which holding more than 90 % of the maximum torque

The torque waveform was obtained from the measurement (Fig. 2). The data from the load cell were transmitted to a computer through a transducer. No filters were used during the torque data acquisition process. The maximum torque was chosen for the data analysis. The wrist torque ratio (=extension/flexion) was calculated for each position. In addition, the agility and the endurance were evaluated. The agility of the muscle strength was defined as the initial slope of the torque/time curve from 25 to 75 % of the maximum torque (Nm/ms) (Fig. 2-b). The endurance was evaluated by the time (s) and the slope (Nm/ms) of the waveform period which holding more than 90 % of the maximum torque (Fig. 2-c). The average of the results of two measurements was used for the comparison among the positions.

### Statistical analysis

The results are expressed as mean ± standard deviation. To test the reliability of the measurements, the intra-class correlation coefficients between first and second measurements were evaluated for the maximum torque. The effects of different forearm positions were compared using a mixed linear model. The effects of the forearm positions were considered to be fixed effects, and the inter-individual variation was considered to be a random effect. Separate models were used for the maximum torque, torque ratio, endurance, and agility data. P values of <0.05 were considered significant. A post hoc pairwise comparison was adapted using Scheffe’s test criteria for the three combinations of the forearm positions. All analyses were performed using Excel Statistics 2012 (SSRI Co., Tokyo, Japan) and SPSS Statistics (IBM, Tokyo, Japan) software.

## Results

**Fig. 3:**
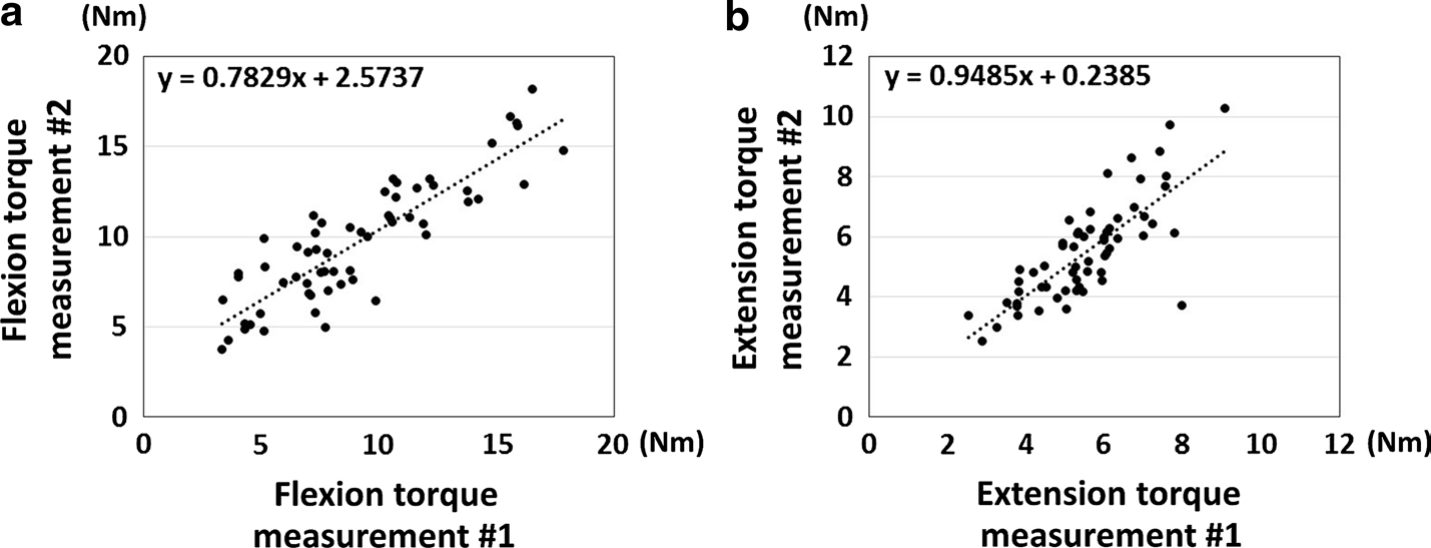
Results of correlations between first and second measurement. **a** Results of the correlations for the wrist flexion torque. **b** Results of the correlations for the wrist extension torque

The results of correlation for the first and second torque measurements are shown in Fig. 3. The intra-class correlation coefficients between first and second measurements were 0.928 and 0.866 for the wrist flexion and extension measurements, respectively.

**Fig. 4:**
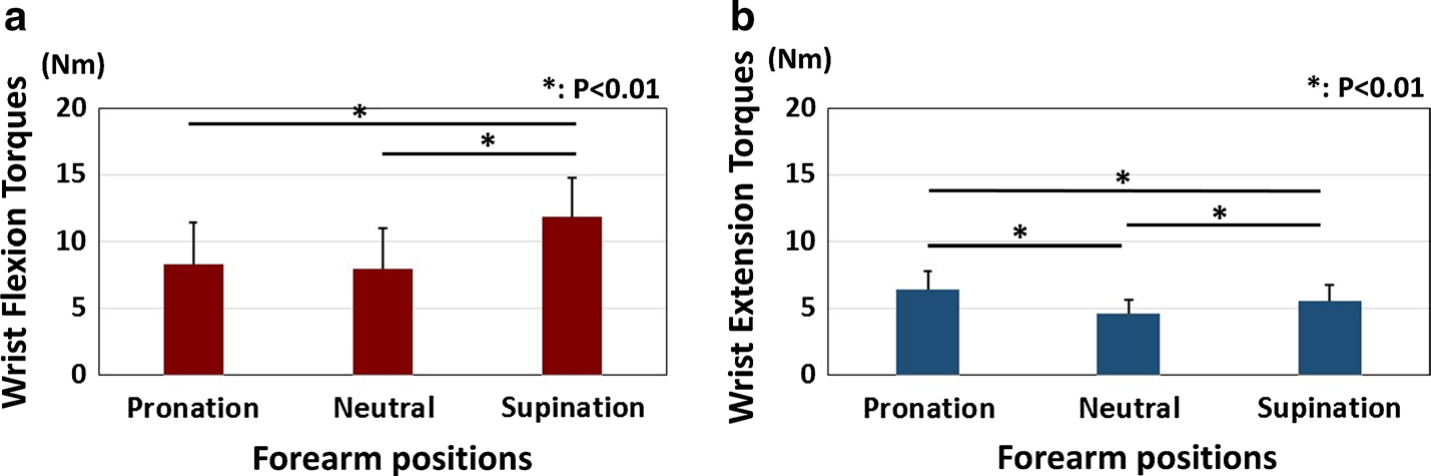
Results of wrist torque. **a** Results of the wrist flexion torques. *Asterisk* showed significant differences between the pronation and the supination positions, and between the neutral and the supination positions (P < 0.01). **b** Results of the wrist extension torques. *Asterisk* showed significant differences among all of the positions (P < 0.01)

**Fig. 5:**
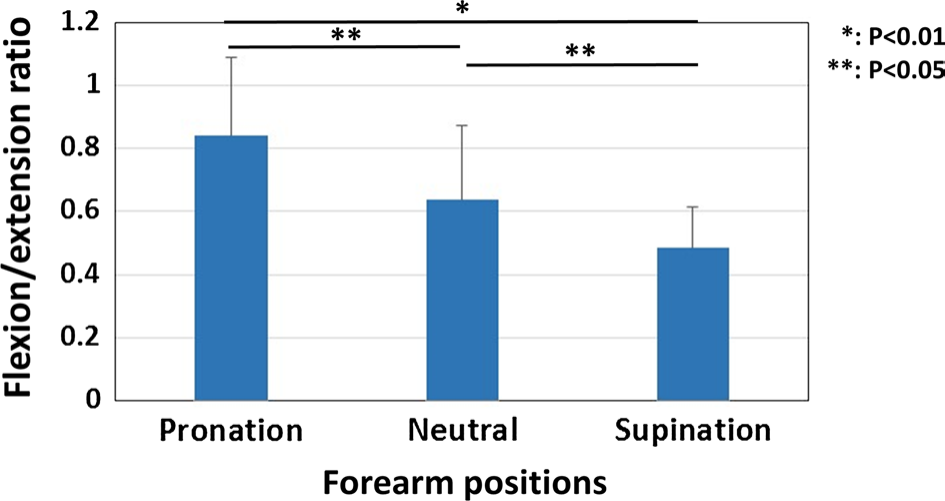
Results of extension/flexion ratio. *Asterisk* showed significant difference between the pronation and supination positions (P < 0.01). *Double* *asterisk* showed significant differences between the pronation and neutral, and between the neutral and supination positions (P < 0.05)

The results of maximum wrist flexion and extension torques are shown in Fig. 4. The maximum wrist flexion torques were 8.0 ± 3.0, 8.3 ± 3.1, and 11.9 ± 2.9 Nm in the neutral, pronation, and supination positions, respectively. The maximum wrist flexion torque was significantly higher when the forearm was in a supination position than when it was in a pronation or neutral position (P < 0.01). There was no significant difference between neutral and pronation positions. The maximum wrist extension torques were 4.6 ± 1.0, 6.5 ± 1.4, and 5.5 ± 1.2 Nm in the neutral, pronation, and supination positions, respectively. The maximum wrist extension torque was significantly higher when the forearm was in the pronation position than when it was in the supination or neutral position (P < 0.01). The results of extension/flexion ratios are shown in Fig. 5. There were significant differences among all of the positions. The lowest ratio was observed in the supination position.

The results of agility and endurance are shown in Table 1. For the wrist flexion agility, there was a significant difference between pronation and supination positions (P < 0.01). For the wrist extension agility, there was a higher value in the pronation position compared to the neutral position, but it was not significant level (P = 0.07). There were no significant differences in the results of endurance.

**Table 1:** Results of agility and endurance

	Agility (Nm/ms)	Endurance time (s)	Slope (Nm/ms)
Wrist flexion			
Neutral	0.026 ± 0.018	2.58 ± 1.21	−3.01 ± 7.66 × 10^−4^
Pronation	0.020 ± 0.011*	2.28 ± 0.95	−1.42 ± 6.95 × 10^−4^
Supination	0.036 ± 0.018*	2.82 ± 1.07	−3.42 ± 4.66 × 10^−4^
Wrist extension			
Neutral	0.019 ± 0.012	2.19 ± 1.35	−4.74 ± 17.6 × 10^−4^
Pronation	0.024 ± 0.015	2.54 ± 0.99	−0.92 ± 5.14 × 10^−4^
Supination	0.021 ± 0.014	2.56 ± 1.15	−0.20 ± 3.49 × 10^−4^

* Showed significant difference between the pronation position and the supination position (P < 0.01)

## Discussion

The wrist flexion and extension torques generated in different forearm positions were investigated in healthy subjects. In addition, the reliability of the wrist torque measurements was evaluated. As a general rule, intra-class correlation coefficients over 0.75 is considered good, and over 0.9 is considered excellent [9]. Therefore, in this study, we could confirm excellent and good reliability for the wrist flexion and extension torque measurements using the apparatus. In addition, it was found that the maximum torque for the wrist flexion was highest in the supination position. The maximum torque for the wrist extension was highest in the pronation position. There was a higher flexion/extension ratio in the supination position compared to the other positions.

The maximum isometric wrist torques in specific postures were reported in several studies [10–13]. The maximum flexion torque ranged from 5 to 20 Nm, and the maximum extension torque ranged from 3 to 14 Nm. The maximum torque values for wrist flexion and extension obtained in this study were similar to those described in previous studies. However, there were little information for the effect of pronation and supination to the wrist torque. Pronation and supination of the forearm are indispensable for activities of daily living. Because of two joints uniting the radius and ulna, the proximal and distal radioulnar joints, the radius can rotate around the ulna [11]. With two bones turning around themselves, it is possible to set muscles, nerves and vessels at the forearm without any risk of twisting. Although the biomechanics of the wrist and elbow have been studied to a considerable extent, the matter of forearm rotation has been discussed relatively few in the wrist torque generation. The described method allowed us to evaluate normal balance of the wrist extension–flexion torques in the different forearm positions. Information about the biomechanical effects of forearm positions is essential for obtaining a better understanding of torque output in normal conditions. In addition, comparing the results of the present study with those obtained in pathological conditions might increase our understanding of the pathophysiology of epicondylitis.

Muscle force generation is influenced by sarcomere length [14] and tendon behavior [15]. Moment arm is determined by the line of muscle–tendon unit force and the center of joint rotation. Alterations in either muscle force or moment arm affect torque output. There are five wrist moving tendons: extensor carpi radialis longus (ECRL), extensor carpi radialis brevis (ECRB), extensor carpi ulnaris (ECU), flexor carpi radialis (FCR), and flexor carpi ulnaris (FCU). The moment contribution of five muscles to the wrist joint was estimated by multiplying the moment arm of the muscle by its physiologic cross sectional area [16, 17]. It is known that the prime working muscles are the FCR and FCU for the wrist flexion, and the ECRB for the wrist extension. Extensor torque was primarily dependent on the moment arm-joint angle relation while flexor torque was influenced by muscle architecture and tendon compliance [18]. Although the forearm rotation didn’t affect the flexor tendon excursions [19], the moment arms for the FCR was largest in the forearm supination position [20]. It is well recognized that the greater the moment arm, the larger is the mechanical advantage of the tendon in providing or resisting specific joint motion. Since the flexor torque profiles were enlarged with the moment arm, there was the highest torque for the wrist flexion in forearm supination position. On the other hand, the wrist extensor torque was dominated by ECRB, given its largest moment arm and greatest force generated capacity [18]. Although the isometric muscle force of ECU was comparable, the poor mechanical advantage limited its functional contribution as a wrist extensor. It was observed that the extensor retinaculum maintains a consistent relationship of the wrist extensors to the rotation axes during the forearm rotation. Thus, the moment arm for the extensor muscles were not significantly changed with forearm rotation [19]. However, it was observed that the ECRB muscle was stretched and worked more effectively for the wrist extension when the forearm pronated [21]. As the results, the wrist extension torque was highest in the pronation position. In addition to the effects of these wrist-moving muscles, the extrinsic finger muscles might also affect wrist torque. It has been demonstrated that the moment-generating capacity of the extrinsic finger muscles has an impact on wrist torque [8, 22]. As it was difficult to separate the contributions of the extrinsic finger muscles from those of the wrist-moving muscles in this in vivo study, this issue requires further consideration in future studies.

Another factor, that may affect the wrist torques, is the positional relation of the bones. The motion of the distal radio-ulnar joint is complicated. The radial epiphysis turns around the ulnar head, whereas the ulnar is moving on a circular path, without any rotation itself [10]. This motion combines extension and lateral translation. In addition, the condyles of the carpal bones are not firmly embedded in the articular surface of the radius. When the hand is fixed on the object, the relative location of the radius and carpus can be changed with forearm supination and pronation. There was another study which showed the rotational dissociation of the radio-ulna and metacarpal bone axes during the forearm rotation. It was found that the rotatory shifting of the carpus at the wrist joint was 45 degree, however it was only 15 degree at the metacarpal level [14]. As the results, the vector sum for the ECR and ECU force exertion direction gets closer to the direction of the wrist extension in the pronation position. This may relate to the higher torque generation for the wrist extension in the forearm pronation position.

It is important to analyze both agility and endurance because these parameters represent different aspects of muscle ability. Agility involves muscle strength, coordination, and neurological function, whereas endurance is the ability to sustain submaximal activity for extended periods of time and resist fatigue. Agility and endurance also indicate how well the fast or slow twitch muscles in the forearms are developed. Therefore, it is important to define normal values for these parameters. It is reasonable to assume that the endurance levels of healthy people will not differ much over short periods of time, such as the 5-s measurement period employed in this study. We chose to limit the study to normal subjects so that we could investigate the normal mechanics of wrist torques in detail before trying to investigate abnormal conditions. Based on these preliminary data, we are planning to measure wrist torque in epicondylitis patients. Such patients might not be able to generate wrist torque for 5 s. Thus, we consider that the measurement period chosen for this study was appropriate. Interestingly, the highest wrist flexion torque and extension agility values were obtained in the supination and pronation positions, respectively. The data collected in this study has provided useful baseline data for future studies.

The strength of our study is that we developed a practical method to evaluate wrist torques in different forearm positions. The diagnosis of lateral and medial epicondylitis has usually been based on local pain at the elbow, tenderness at the epicondyle on palpation, and pain at the epicondyle on resisted isometric extension or flexion of the wrist. The repeatability of the isometric and palpation tests has been moderate [23]. Since it was suggested that work tasks demanding forceful activities were associated with a higher risk of epicondylitis [5, 24, 25], it may be necessary to estimate how much load applied to the tendon insertion. The method which showed in this study is a way to evaluate the relation between loading factors and epicondylitis. In addition, the normal balance of wrist flexion and extension torques were characterized in this study. It may be possible to advocate a new diagnostic criteria for the epicondylitis with comparing these data to the patients. According to the excellent repeatability of the measurement, we will evaluate this in the epicondylitis patients in the future study.

The limitations of this study should be noted. First, we did not make any attempt to study the effect of isokinetic or isotonic wrist torque. It was demonstrated that different torques were observed during isokinetic contraction compared to isometric contraction [26]. Thus, the characteristics of the wrist torque may differ with differential muscle contractions. Second, we didn’t measure the wrist torque in different wrist positions. Previous studies demonstrated that maximum muscle force and peak joint torque occur at different wrist joint angles [27–29]. In addition, moment arms varied considerably throughout the range of wrist joint motion [21]. The wrist torques may be different in different joint angle of the wrist.

## Conclusions

The normal balance of the wrist flexion–extension torques in different forearm positions were characterized. The highest torque for the wrist flexion was observed in the supination position. The highest torque for the wrist extension was observed in the pronation position. There was a higher flexion/extension ratio in the supination position compared to the other positions. The apparatus used in this study will serve as a potential tool in the assessment of epicondylitis, clinically. The results of this study can provide basic knowledge to assess biomechanical effects of forearm positions on the entheses.
